# αvβ3 Integrin Expression and Mitogenic Effects by Thyroid Hormones in Chronic Lymphocytic Leukemia

**DOI:** 10.3390/jcm10081766

**Published:** 2021-04-19

**Authors:** Uri Abadi, Avivit Weisz, Dvora Kidron, Aviva Katzav, Aleck Hercbergs, Paul J. Davis, Martin H. Ellis, Osnat Ashur-Fabian

**Affiliations:** 1 Translational Hemato-Oncology Laboratory, Hematology Institute and Blood Bank Meir Medical Center, Kfar-Saba 44821, Israel; Uri.Abadi@clalit.org.il (U.A.); avivitweisz@gmail.com (A.W.); martinel@clalit.org.il (M.H.E.); 2 Sackler Faculty of Medicine, Tel Aviv University, Tel Aviv 69978, Israel; 3 Department of Human Molecular Genetics and Biochemistry, Sackler Faculty of Medicine, Tel Aviv University, Tel Aviv 69978, Israel; 4 Department of Pathology, Meir Medical Center, Kfar Saba 44821, Israel; dkidron@clalit.org.il (D.K.); aviva.katzav@clalit.org.il (A.K.); 5 Radiation Oncology, Cleveland Clinic, Cleveland, OH 44195, USA; hercbergs@gmail.com; 6 Department of Medicine, Albany Medical College, Albany, NY 12208, USA; pdavis.ordwayst@gmail.com

**Keywords:** chronic lymphocytic leukemia, αvβ3 integrin, thyroid hormone

## Abstract

Background: Chronic lymphocytic leukemia (CLL) is the most common adult leukemia. The thyroid hormones, T3 and T4, bind the αvβ3 integrin and activate phosphorylates ERK (pERK). These tumor-promoting actions were reported in a number of malignancies, but not in CLL. Methods: Primary cells from 22 CLL patients were verified for disease markers (CD5/CD19/CD23) and analyzed for αvβ3 by flow cytometry (FC), ImageStream, Western blots (WB), and immunohistochemistry (IHC) in archival bone marrow (BM, *n* = 6) and lymph node (LN, *n* = 5) tissues. Selected samples (*n* = 8) were incubated with T3 (1–100 nM) or T4 (0.1–10 µM) for 30 min, and the expression levels of αvβ3, pERK and PCNA (cell proliferation marker) were determined (WB). Results: αvβ3 was detected on the membrane of circulating CLL cells and in the BM but not in the LN. T3 and T4 enhanced αvβ3 protein levels in primary CLL cells. Similarly, pERK and PCNA were rapidly induced in response to T3 and T4 exposure. Conclusions: αvβ3 integrin is expressed on primary CLL cells and is induced by thyroid hormones. We further suggest that the hormones are mitogenic in these cells, presumably via αvβ3-mediated signaling.

## 1. Introduction

Leukemia is a cancer of blood or bone marrow, characterized by accumulation of functionally incompetent malignant cells, which interfere with normal cell production [1]. Chronic lymphocytic leukemia (CLL) is the most common type of adult leukemia, characterized by accumulation of mature lymphocytes in the peripheral blood, lymph nodes, and bone marrow. CLL cells express a distinct immunophenotype, characterized by coexpression of CD19, CD5, and CD23 [2]. Although originally CLL was viewed as a tumor caused by the accumulation of long-lived but mainly resting lymphocytes, experiments conducted in recent years have shown that CLL contains a small fraction of actively proliferating cells [3]. CLL has a variable clinical course and prognosis, ranging from an asymptomatic process that does not necessitate intervention, to a progressive, fatal illness. Interactions between the malignant cells and the microenvironment, mainly in the lymph nodes, are highly important in CLL’s pathogenesis and control proliferation and survival [4].

Integrins are transmembrane glycoproteins, which facilitate cell–cell and cell–extracellular matrix (ECM) adhesion as well as cell migration. They are a family of cell surface receptors and are present in all nucleated cells. The mammalian integrins are assembled from 18-α subunits and 8-β subunits, and include receptors for the major ECM proteins. In combination, they form at least 24 different heterodimers. Although integrins were originally discovered as adhesion molecules, they also act as true signaling receptors, activating signals necessary to support cell proliferation, migration, viability, and angiogenesis. An important member of this family is the αvβ3 integrin, which participates in many essential cancer signaling pathways. αvβ3 is overexpressed in an array of cancer cells and was found to correlate with tumor progression [5,6].

The thyroid hormones (TH), thyroxine (T4) and triiodothyronine (T3), are mostly recognized for their important role in normal growth, development, and metabolism. However, several lines of evidence have also suggested tumor-promoting effects by these hormones [7]. In the past decade, the mechanism underlying the tumor-promoting actions of thyroid hormones has been elucidated. It is probably not T3 or its classical genomic action via the thyroid hormone receptors, TRα or TRβ, that promotes tumor growth. Rather, these tumor growth-promoting activities are mediated by so-called nongenomic signaling, which is predominantly initiated by T4 that binds with high affinity to a plasma membrane integrin, αvβ3 [8]. This T4-responsive integrin is overexpressed in many tumor cells and tumor vasculature. αvβ3 integrin transmits the T4 signals into the cell and leads to activation of an array of mitogenic signaling pathways, leading to regulation of gene programs involved in cellular proliferation. The molecular receptor for thyroid hormone upon the integrin αvβ3 was discovered at close proximity to the RGD recognition site. Three-dimensional crystallographic modelling [9] and mathematical modelling of the kinetics of thyroid hormone binding [10] revealed that T3 and T4 bind to an independent site at the interface of the αv and β3 domains and initiate various mitogenic outcomes. While T4 binds and initiates mitogenic effects at physiological concentrations, T3 is less potent and mediates these actions only at supraphysiological level. In this work, we demonstrate that αvβ3 integrin is expressed on primary CLL cells and that thyroid hormones induce the expression of αvβ3, as well as of MAPK signaling, suggesting a mitogenic role, presumably via αvβ3-mediated signaling.

## 2. Experimental Section

Sample collection. Peripheral blood (PB) was collected from CLL patients (*n* = 22) and healthy controls (*n* = 3) upon signing informed consent. This study was approved by the Institutional Review Board and ethics committee of The Meir Medical Center. Clinical parameters regarding CLL disease characteristics were collected from the patient’s medical record and are presented in Table S1. Mononuclear cells were isolated by Ficoll-Paque gradient centrifugation according to the manufacturer’s instructions (Sigma-Aldrich, St. Louis, MO, USA). The cells were immediately immunophenotyped for CLL markers (CD5/CD19/CD23) and αvβ3 expression by flow cytometry and seeded for the various experiments detailed below. For some samples, the in vitro assays were not conducted due to limited materials transferred from the routine laboratory.

Reagents and antibodies. Triodothyronine (T3) and thyroxine (T4) were obtained from Sigma-Aldrich (Steinheim, Germany) and dissolved in DMSO to 100 mM, followed by dissolving to 1 mM in KOH-propylene glycol (final concentration of 0.04 N KOH with 0.4% polyethylene glycol (vol/vol)). Primary antibodies against PCNA (SC-7907) and β3 integrin (SC-14009) were from Santa Cruz Biotechnology (Dallas, TX, USA). Anti ERK1/2, phosphoERK1/2, and GAPDH (used for protein-loading normalization) were from Cell Signaling Technology (Leiden, The Netherlands). For IHC, rabbit anti-human αvβ3 polyclonal antibody was used (Abbiotec, San-Diego, CA, USA). Monoclonal antibody against αvβ3 integrin (LM609, FITC-conjugated) was from Merck Millipore (Darmstadt, Germany). Monoclonal murine antibodies CD5 (FITC-conjugated) and CD19 (PE-conjugated) were from IQProducts, Groningen, the Netherlands. Anti-CD23 (PE-conjugated) was purchased from DakoCytomation (Glostrup, Denmark).

Flow cytometry analysis (MACSQant, Miltenyi Biotec). The cells (10^5^ cells) were immunophenotyped by using 10 µg/mL FITC-CD5, PE-CD19, PE-CD23, or FITC-αvβ3 (clone LM609) antibodies.

ImageStream technology. An Amnis ImageStream^®^X Mark II multispectral imaging flow cytometer (Amnis Corporation, Seattle, WA, USA) was used. For αvβ3 membrane expression, 1 × 10^6^ cells were incubated with 10 µg/mL PE-labeled αvβ3 antibody (LM-609, Millipore), fixed and permeabilized using FIX & PERM^®^ Cell Fixation & Cell Permeabilization Kit (Life technologies). IgG was used as negative isotype control. Nucleus is stained blue with Hoechst (33342, molecular probes, Eugene, OR, USA).

*Immunohistochemistry (IHC) from FFPE sections.* A retrospective analysis was performed on archival formalin-fixed, paraffin-embedded (FFPE) tissue blocks at the Histopathology Department, Meir Medical Center. Four-micron sections of FFPE tissue blocks of bone marrows (BM) and lymph nodes (LN) were cut. The immunostains were performed following manufacturers protocols, on Ventana Autostainer (Ventana Medical Systems, Inc., Tucson, AZ, USA). Optimal 1:200 dilution of αvβ3 antibody was determined following calibration with 1:100, 1:200, or 1:400 dilutions. Staining with rabbit IgG served as negative control in all samples. The slides were assessed by a board-certified hematopathologist. Slides were imaged using the Aperio VERSA SlideScanner at X20 and X40 magnifications.

Western blotting. CLL cells were seeded (20 × 10^6^ cells/24-well plates) in RPMI medium in the absence of serum and treated with increasing doses of T3 (1–100 nM) or T4 (100 nM and 1–10 µM) for 30 min. Whole-cell proteins were extracted and separated on 10–12.5% polyacrylamide gels, fast-transferred to PVDF membranes, incubated with antibodies against pERK (normalized to ERK), β3 integrin, or PCNA (normalized to actin or GAPDH), and visualized using horseradish peroxidase (HRP)-conjugated secondary antibody (1:10,000, Jackson Immuno Research Laboratories, West Grove, PA, USA) followed by enhanced chemiluminescence (ECL) detection (Biological Industries, Beit Haemek, Israel). Integrated optical densities of the bands were measured by Image reader Las3000, Multi-gauge v3.0 software.

Statistical analysis. Experiments were analyzed by a Student’s unpaired t-test for significance or ANOVA for multiple comparisons (*p* < 0.05).

## 3. Results

### 3.1. αvβ3 Integrin Is Expressed in Circulating CLL B Lymphocytes and in the Bone Marrow but Not in the Lymph Node of CLL Patients

**Figure 1:**
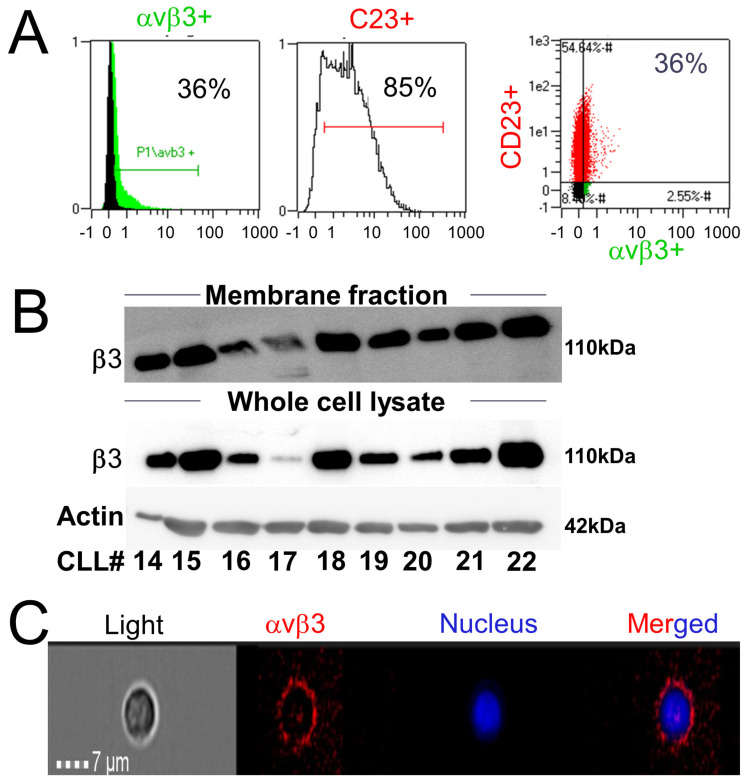
αvβ3 integrin is expressed on the membrane of primary CLL B lymphocytes. (**A**) A representative flow cytometry analysis of αvβ3 (FITC-labeled) and CD23 (PE-labeled) in cells from CLL#1. (**B**) Western blot analysis of αvβ3 integrin in membrane proteins and whole-cell lysates of CLL cells (CLL#14-22). Representative blot of two repeats is presented. (**C**) ImageStream analysis of membrane αvβ3 (PE) in a representative CLL B lymphocyte from CLL#1. The nucleus was stained blue with Hoechst; 20× magnification images of separate and merged channels are shown.

Mononuclear cells from the PB of twenty-two CLL patients (CLL#1-22) were collected and confirmed for B lymphocyte CLL markers (CD5/CD19). In all samples, the majority of peripheral B lymphocytes were monoclonal CD5/CD19-positive cells. The percentage of CD5/CD19-positive cells from total lymphocyte counts and the relevant clinical data for the study cohort are shown in Table S1. Next, the collected cells were co-stained with an antibody against the αvβ3 integrin (FITC-conjugated) and an antibody against CD23 (PE-conjugated), another common B lymphocytes CLL marker. A consistent αvβ3 expression was observed in 10–36% of gated cells of the study population (Figure S1). The mean fluorescence intensity (MFI) of αvβ3, as fold from a negative isotype control, ranged between 1.2 and 8-fold (average 4.8 ± 4.8), with an average 30.6 ± 8.4% of cells expressing the integrin, indicating diversity in the extent of expression between samples (Table S1). A representative sample with integrin positivity in 36% of cells (Figure 1A) confirmed αvβ3 presence exclusively on the surface of CD23+ B lymphocytes. Similar results were obtained in thirteen additional samples (Figure S2). A parallel analysis indicated that normal B lymphocytes are αvβ3-integrin-negative (Figure S3), in accordance with previous reports [11]. Western blots on proteins extracted from both membrane fractions and whole-cell lysates of primary CLL cells further established positive membrane expression of αvβ3 (Figure 1B). Similar to the flow cytometry results, we observed a varied expression between patients. A classical membrane staining on a representative B lymphocyte is shown using the ImageStream technology, which combines flow cytometry with fluorescent microscopy (Figure 1C). Taken together, these collective results indicated that a fraction of malignant CLL B lymphocytes express membrane αvβ3 integrin.

**Figure 2:**
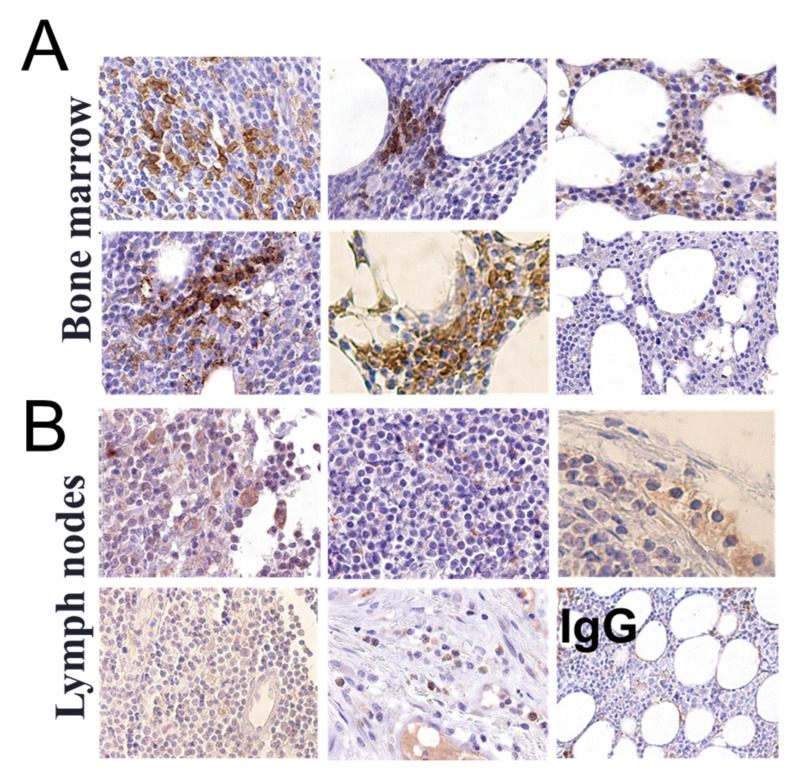
Immunohistochemistry for αvβ3 integrin in the bone marrow and lymph node of CLL patients. Four-micron sections of FFPE tissue blocks of (**A**) bone marrows and (**B**) lymph nodes were immune-stained with αvβ3 antibody. Staining with rabbit IgG served as negative control. Slides were imaged using the Aperio VERSA SlideScanner at 20× magnification.

We next assessed whether αvβ3 is expressed not only in circulating CLL cells, but also within the bone marrow (BM) and lymph nodes (LN) of patients. For that, archived formalin-fixed, paraffin-embedded (FFPE) tissue sections were collected from six BM and five LN samples and analyzed by immunohistochemistry (IHC) using anti-human αvβ3 antibody. IgG isotype control was used as negative control. Five BM samples displayed integrin staining in restricted regions, mainly in lymphocytes (Figure 2A), while one sample (CLL#4) was αvβ3-negative, although circulating CLL cells from this patient were integrin-positive. All LN samples stained negative for αvβ3 integrin (Figure 2B). These results suggest that αvβ3 integrin is expressed on circulating CLL lymphocytes but also within the BM niche.

### 3.2. Thyroid Hormones Induce αvβ3 Integrin Expression in Primary CLL Cells

**Figure 3:**
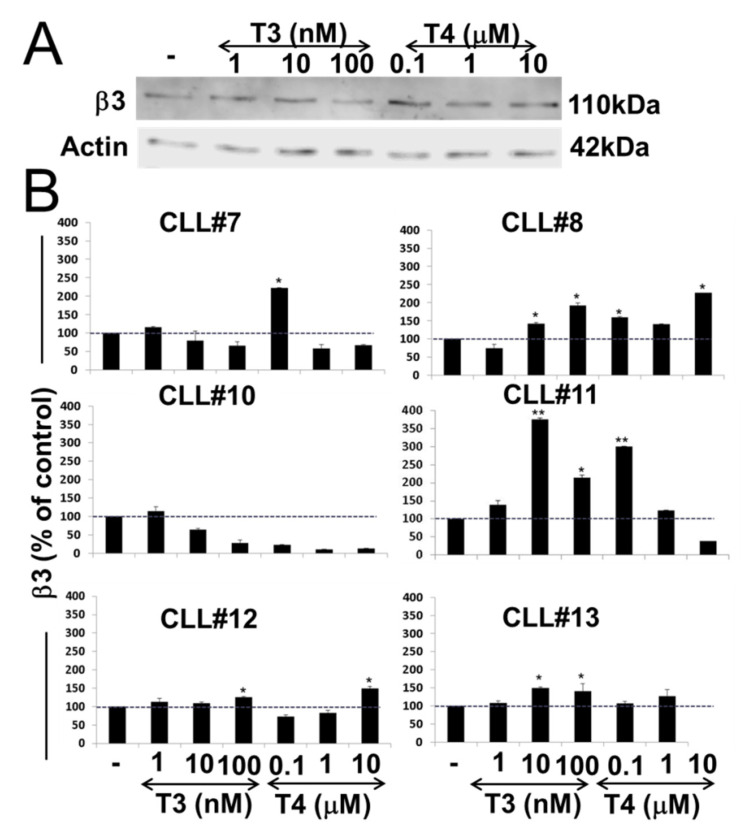
T3/T4-induced β3 protein level in primary CLL cells. (**A**) Western blots analyses of β3 protein levels in whole-cell lysates of a representative CLL sample (CLL#7) after 30 min T3/T4 treatments over a dose range. Representative blot of two repeats is presented. (**B**) Respective quantification of β3 protein levels in six CLL samples, normalized to loading control, is presented as % of vehicle control. Significance from vehicle control is indicated by * *p* < 0.05, ** *p* < 0.005.

The thyroid hormones, T3 and T4, were shown to act as growth factors in numerous cancer models via binding to the αvβ3 integrin [8]. However, limited data exist on the role of these hormones in hematological malignancies, and no study was conducted to date on CLL, the most common adult leukemia. Our observation that primary CLL cells express the αvβ3 integrin laid the basis for such an examination. First, we aimed to study whether in CLL, similar to other tumor models, treatment thyroid hormones induce the protein level of αvβ3 integrin receptor. To that end, we isolated primary cells from six CLL patients and grew the cells (20 × 10^6^ cells/24 wells) in the absence or presence of thyroid hormones. To control for hormone concentrations, the cells were first incubated overnight in the presence of hormone-deprived serum, followed by treatments with increasing concentrations of T3 (1–100 nM) or T4 (0.1–10 µM). After 30 min of incubation, total proteins were extracted and the level of β3 integrin monomer was assessed by immunoblots. The integrin level in a representative sample is presented in Figure 3A, and quantification in all examined samples is shown in Figure 3B. Western blots for the six patient samples are presented in Figure S4. Results indicate that T3 and T4 quickly induced the protein levels of the β3 integrin in five of the samples. The integrin expression was uniquely affected by T3 or T4, by different hormone concentrations and resulted in different protein induction levels, which were not dose-dependent. In cells from a single patient (CLL#10), an opposite trend was documented, with integrin inhibition following T3 and T4 treatments. This diverse effect may be related to the fact that the integrin expression in this specific sample was the lowest in the study group, suggesting that initiating a biological response by the hormones may require a minimal αvβ3 threshold.

### 3.3. Mitogenic Signals Are Induced by Thyroid Hormones in Primary CLL Cells

**Figure 4:**
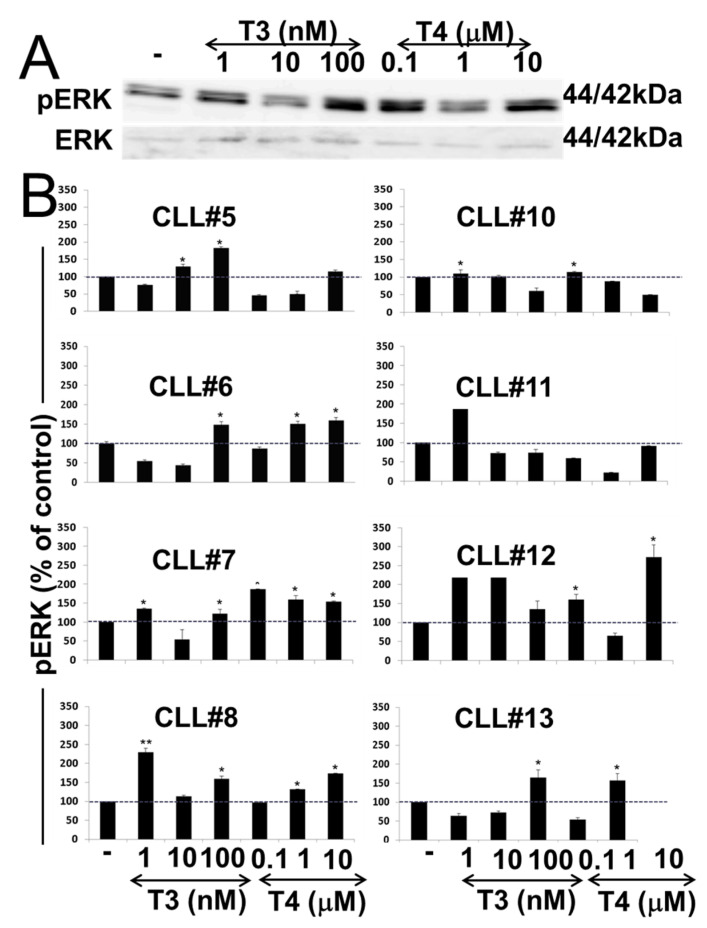
T3/T4-induced ERK activation in CLL cells. (**A**) Western blots analyses of pERK protein levels were performed on whole-cell lysates of CLL cells of a representative CLL sample (CLL#7) after 30 min T3/T4 treatments over a dose range. Representative blot of two repeats is presented. (**B**) Respective quantification of pERK protein levels in eight CLL samples normalized to total ERK is presented as % of vehicle control. Significance from vehicle control is indicated by * *p* < 0.05, ** *p* < 0.005.

The activation of ERK has been closely correlated with the thyroid hormones’ action in various cancer cells. We therefore aimed to study the effect of both thyroid hormones on the phosphorylation (activation) of extracellular regulated kinase (ERK). CLL cells from eight patients were isolated, seeded (20 × 10^6^ cells/24 wells) overnight in serum-free conditions, and treated for 30 min with a dose range of T3 (1–100 nM) or T4 (0.1–10 µM). Total proteins were extracted, and the level of pERK was evaluated by immunoblots. pERK levels in a representative CLL sample are presented in Figure 4A, and the respective quantification in all examined samples is shown in Figure 4B. Western blots for the entire patient samples are presented in Figure S5. Western blot results indicate that both T3 and T4 affected pERK activation in a manner that was patient-specific and hormone-specific. In some of the samples, a significant induction was observed by only one of the hormones (e.g., CLL#5 by T3), and by both hormones (e.g., CLL#12), while cells from CLL#10, in accord with the low integrin expression level and the limited effect on its expression, as shown before, were less sensitive to the hormonal effects.

**Figure 5:**
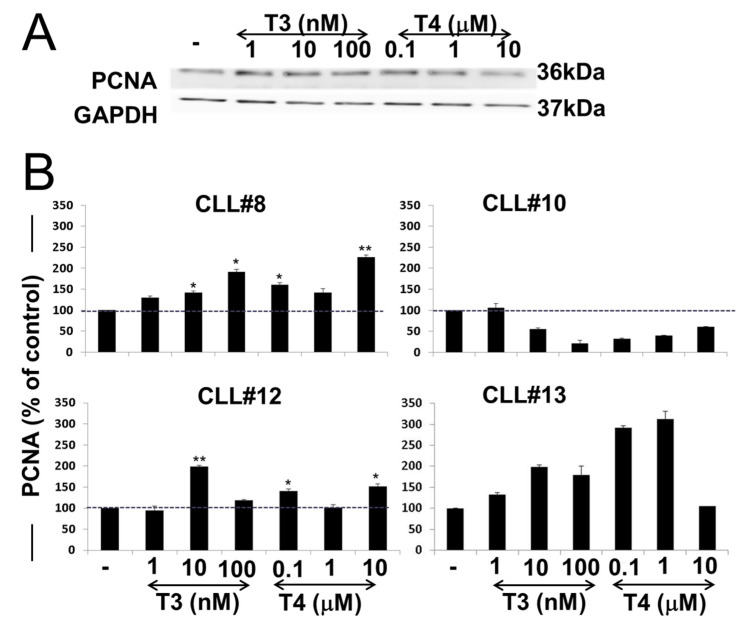
T3/T4-induced PCNA activation in CLL cells. (**A**) Western blots analyses of PCNA protein levels were performed on whole-cell lysates of representative CLL cells (CLL#8) after 30 min T3/T4 treatments over a dose range. Representative blot of two repeats from each patient’s sample is presented. (**B**) Respective quantification of PCNA protein levels, normalized to loading control, is presented in the four CLL samples as % of vehicle control. Significance from vehicle control is indicated by * *p* < 0.05, ** *p* < 0.005.

The same experiment was next evaluated for proliferating cell nuclear antigen (PCNA), a common proliferation marker. PCNA level in a representative sample is presented in Figure 5A, and the respective quantification of the examined samples is shown in Figure 5B. Western blots for the remaining samples are shown in Figure S6. In three out of the four samples examined, PCNA was quickly induced by both T3 and T4, while in cells derived from CLL#10, which exhibited a very low integrin level, an opposite trend was documented. These collective results suggest that each CLL patient has different sensitivity towards thyroid hormones’ signaling pathways.

## 4. Discussion

The integrin αvβ3 has been under rigorous investigation in cancer due to its key roles in disease progression and invasion [12]. This integrin is amply expressed on the plasma membrane of an array of cancer cells, including of hematological malignancies. The expression and involvement of integrins were shown before in CLL [13,14,15,16,17], the most common adult leukemia, however, specific data on αvβ3 integrin expression and function in this disease are limited.

In this work, we have provided, by several complementary methods, evidence that in contrast to the integrin levels in normal lymphocytes, αvβ3 integrin is expressed on the membrane of circulating B lymphocytes from CLL patients. These results correspond with reports on low levels of αv integrins in primary normal B lymphocytes [11] and positive αvβ3 expression in B-cells from CLL patients [18,19]. The integrin expression was heterogeneous between patient samples, with regards to the fraction of integrin-positive subpopulations and the magnitude of the integrin expression, in accord with previous published results [20]. The integrin expression was evident in CD23+ cells, a common CLL marker that promotes the activation and proliferation of normal B lymphocytes and has an important role in the process of malignant transformation in B-CLL [21,22,23]. Notably, CD23 is also considered a ligand for αvβ3. The identification of αvβ3 in CLL cells may propose a prognostic role in this disease. A previous report indicated that patients whose cells did not express β integrin chains (β1–3), fell into the most favorable prognostic group, with lower lymphocytosis and the absence of splenomegaly, diffuse bone marrow infiltration, and therapy requirement [20]. In addition, αvβ3 was shown to enhance cell invasion in human multiple myeloma [24] and to be involved in proliferation and migration of lymphoid tumor cell lines [25]. Other studies have linked the integrin expression in CLL cells and clinical outcome of the disease [15,16,17,20].

Examination of the integrin expression in the bone marrow of CLL patients identified focal integrin staining, while no integrin was evident in lymph node samples. Our previous work further demonstrated that αvβ3 integrin is completely absent in normal BM [26]. As integrin-mediated adhesion was reported to play a central role in trafficking and retention of hematopoietic cells in the BM and lymphoid, abnormalities in αvβ3 expression may also take part in the spread of CLL cells into the BM. However, our results, which are based on a limited number of CLL samples and do not provide a mechanistic basis, should be taken with cautious, and a study on a larger number of patients is merited.

Following the observation of αvβ3 expression in CLL cells and because thyroid hormones were established as ligands for this receptor, initiating a cascade of pro-tumorigenic events [8], we studied the effect of T3 and T4 on these cells. We observed increased integrin abundance in the majority of CLL samples following hormone binding, corresponding with our previous work on myeloma cells [27] and ovarian cancer [28]. We have further shown that both T3 and T4 initiate activation of the MAPK pathway, parallel to increased levels of PCNA, a central proliferation marker. As far as we know, the sole support for our observation that thyroid hormones may possess growth-promoting capabilities in CLL cells is early studies, in which induced hypothyroidism attenuated lymphomatous infiltrations and prolonged survival of mice and rats with transplanted lymphomas [29,30]. Other recent studies presented such a proliferative effect by the hormones–αvβ3 pathway in an array of solid tumor models and hematological malignancies (reviewed in [31]), focusing mainly on lymphoproliferative disorders, such as T-cell lymphomas (Reviewed in [32]).

## 5. Conclusions

To conclude, this work provides the first indication for the existence of the thyroid hormones–αvβ3 axis in CLL cells and suggests the need for mechanism-oriented research in order to elucidate the potential growth-promoting actions initiated via this pathway.

## Data Availability

Source data for all blot images are provided with this paper. All other relevant data are available from the corresponding author upon reasonable request.

## Supplementary Materials

The following are available online at https://www.mdpi.com/article/10.3390/jcm10081766/s1, Table S1: Clinical characteristics of the study cohort. Figure S1: αvβ3 is expressed in circulating cells from CLL patients. Figure S2: αvβ3 is expressed in B lymphocyte cells from CLL patients. Figure S3: αvβ3 is absent in normal B lymphocytes. Figure S4: T3/T4-induced β3 integrin in CLL cells. Figure S5: T3/T4-induced ERK activation in CLL cells. Figure S6: T3/T4-induced PCNA activation in CLL cells.

## References

[1] Hus I., Roliński J. (2015). Current concepts in diagnosis and treatment of chronic lymphocytic leukemia. Contemp. Oncol..

[2] Gaidano G., Foà R., Dalla-Favera R. (2012). Molecular pathogenesis of chronic lymphocytic leukemia. J. Clin. Investig..

[3] Messmer B.T., Messmer D., Allen S.L., Kolitz J.E., Kudalkar P., Cesar D., Murphy E.J., Koduru P., Ferrarini M., Zupo S. (2005). In vivo measurements document the dynamic cellular kinetics of chronic lymphocytic leukemia B cells. J. Clin. Investig..

[4] Cacciatore M., Guarnotta C., Calvaruso M., Sangaletti S., Florena A.M., Franco V., Colombo M.P., Tripodo C. (2012). Microenvironment-centred dynamics in aggressive B-cell lymphomas. Adv. Hematol..

[5] Desgrosellier J.S., Cheresh D.A. (2010). Integrins in cancer: Biological implications and therapeutic opportunities. Nat. Rev. Cancer.

[6] Guo W., Giancotti F. (2004). Integrin signalling during tumour progression. Nat. Rev. Mol. Cell Biol..

[7] Moeller L.C., Fuhrer D. (2013). Thyroid hormone, thyroid hormone receptors, and cancer: A clinical perspective. Endocr. Relat. Cancer.

[8] Davis P.J., Goglia F., Leonard J.L. (2016). Nongenomic actions of thyroid hormone. Nat. Rev. Endocrinol..

[9] Cody V., Davis P.J., Davis F.B. (2007). Molecular modeling of the thyroid hormone interactions with alpha v beta 3 integrin. Steroids.

[10] Lin H.Y., Landersdorfer C.B., London D., Meng R., Lim C.U., Lin C., Lin S., Tang H.Y., Brown D., Van Scoy B. (2011). Pharmacodynamic modeling of anti-cancer activity of tetraiodothyroacetic acid in a perfused cell culture system. PLoS Comput. Biol..

[11] Huang S., Stupack D., Mathias P., Wang Y., Nemerow G. (1997). Growth arrest of Epstein–Barr virus immortalized B lymphocytes by adenovirus-delivered ribozymes. Proc. Natl. Acad. Sci. USA.

[12] Wilder R. (2002). Integrin alpha V beta 3 as a target for treatment of rheumatoid arthritis and related rheumatic diseases. Ann. Rheum. Dis..

[13] Till K.J., Spiller D.G., Harris R.J., Chen H., Zuzel M., Cawley J.C. (2005). CLL, but not normal, B cells are dependent on autocrine VEGF and α4β1 integrin for chemokine-induced motility on and through endothelium. Blood.

[14] Plate J., Long B., Kelkar S. (2000). Role of β2 integrins in the prevention of apoptosis induction in chronic lymphocytic leukemia B cells. Leukemia.

[15] Baldini L.G., Cro L.M. (1994). Structure and function of VLA integrins: Differential expression in B-cell leukemia/lymphoma. Leuk. Lymphoma.

[16] Takeuchi H., Katayama I. (1993). Surface phenotype and adhesion activity of B-cell chronic lymphoid leukemias. Leuk. Lymphoma.

[17] Vincent A., Cawley J., Burthem J. (1996). Integrin function in chronic lymphocytic leukemia. Blood.

[18] Mateo V., Lagneaux L., Bron D., Biron G., Armant M., Delespesse G., Sarfati M. (1999). CD47 ligation induces caspase-independent cell death in chronic lymphocytic leukemia. Nat. Med..

[19] Bairey O., Zimra Y., Rabizadeh E., Shaklai M. (2004). Expression of adhesion molecules on leukemic B cells from chronic lymphocytic leukemia patients with predominantly splenic manifestations. IMAJ-RAMAT GAN.

[20] De Rossi G., Zarcone D., Mauro F., Cerruti G., Tenca C., Puccetti A., Mandelli F., Grossi C.E. (1993). Adhesion molecule expression on B-cell chronic lymphocytic leukemia cells: Malignant cell phenotypes define distinct disease subsets. Blood.

[21] Jurisic V., Colovic N., Kraguljac N., Atkinson H.D., Colovic M. (2008). Analysis of CD23 antigen expression in B-chronic lymphocytic leukaemia and its correlation with clinical parameters. Med. Oncol..

[22] Fournier S., Tran D., Suter U., Biron G., Delespesse G., Sarfati M. (1991). The in vivo expression of type B CD23 mRNA in B-chronic lymphocytic leukemic cells is associated with an abnormally low CD23 upregulation by IL-4: Comparison with their normal cellular counterparts. Leuk. Res..

[23] Sarfati M., Chevret S., Chastang C., Biron G., Stryckmans P., Delespesse G., Binet J.-L., Merle-Beral H., Bron D. (1996). Prognostic importance of serum soluble CD23 level in chronic lymphocytic leukemia. Blood.

[24] Ria R., Vacca A., Ribatti D., Di Raimondo F., Merchionne F., Dammacco F. (2002). Alpha(v)beta(3) integrin engagement enhances cell invasiveness in human multiple MM. Haematologica.

[25] Vacca A., Ria R., Presta M., Ribatti D., Iurlaro M., Merchionne F., Tanghetti E., Dammacco F. (2001). Avb3 integrin engagement modulates cell adhesion, proliferation, and protease secretion in human lymphoid tumor cells. Exp. Hematol..

[26] Cohen K., Abadi U., Hercbergs A., Davis P.J., Ellis M., Ashur-Fabian O. (2018). The induction of myeloma cell death and DNA damage by tetrac, a thyroid hormone derivative. Endocr. Relat. Cancer.

[27] Cohen K., Ellis M., Khoury S., Davis P.J., Hercbergs A., Ashur-Fabian O. (2011). Thyroid hormone is a MAPK-dependent growth factor for human myeloma cells acting via alphavbeta3 integrin. Mol. Cancer Res..

[28] Shinderman-Maman E., Cohen K., Weingarten C., Nabriski D., Twito O., Baraf L., Hercbergs A., Davis P.J., Werner H., Ellis M. (2016). The thyroid hormone-alphavbeta3 integrin axis in ovarian cancer: Regulation of gene transcription and MAPK-dependent proliferation. Oncogene.

[29] Morris D., Wolff F., Upton A.C. (1957). The influence of the thyroid gland on the survival of rats and mice bearing transplanted lymphoid leukemia. Cancer Res..

[30] Morris D.M. (1960). The effect of thyroidectomy and thyroid-stimulating hormone on a transplanted acute leukemia in the Fischer rat. Cancer Res..

[31] Krashin E., Piekiełko-Witkowska A., Ellis M., Ashur-Fabian O. (2019). Thyroid hormones and cancer: A comprehensive review of preclinical and clinical studies. Front. Endocrinol..

[32] Cayrol F., Sterle H.A., Díaz Flaqué M.C., Barreiro Arcos M.L., Cremaschi G.A. (2019). Non-genomic actions of thyroid hormones regulate the growth and angiogenesis of T cell lymphomas. Front. Endocrinol..

