# Supplementary material for: αvβ3 Integrin Expression and Mitogenic Effects by Thyroid Hormones in Chronic Lymphocytic Leukemia
Source: J Clin Med. 2021 Apr 19;10(8):1766. doi: 10.3390/jcm10081766 (PMC8073405; doi:10.3390/jcm10081766)
Supplement: Supplementary file 1 [file jcm-10-01766-s001.zip › Supplementary material Revised 25 March 2021.pdf]

Supplementary material:

αvβ3 integrin expression and mitogenic effects by thyroid hormones in chronic lymphocytic leukemia. Uri Abadi et al.

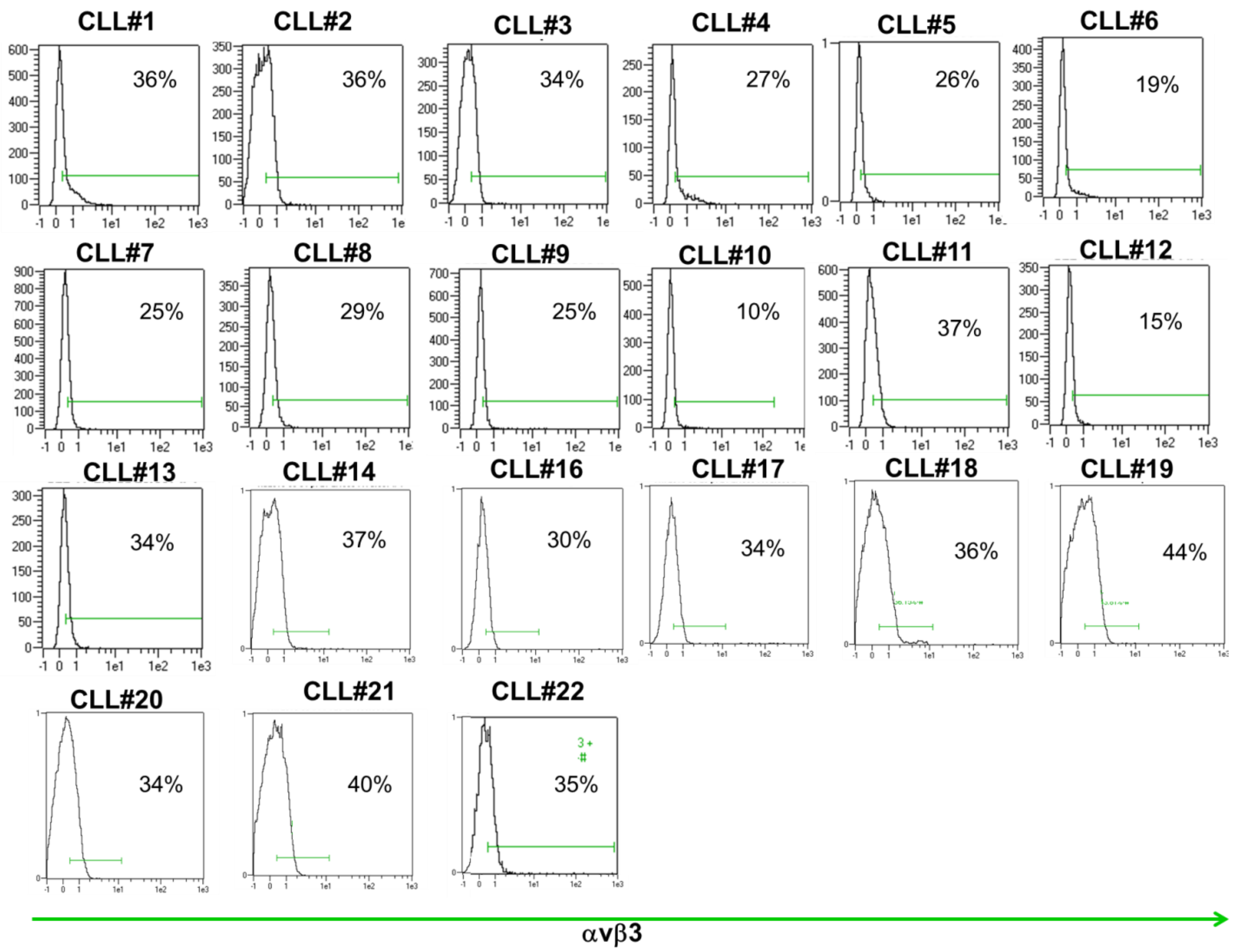

Supplementary Figure S1: αvβ3 is expressed in circulating cells from CLL patients. Flow-cytometry analyses of αvβ3 (FITC-labeled) in cells from the CLL study cohort (n=22). Percentage of integrin positive cells is depicted in each sample

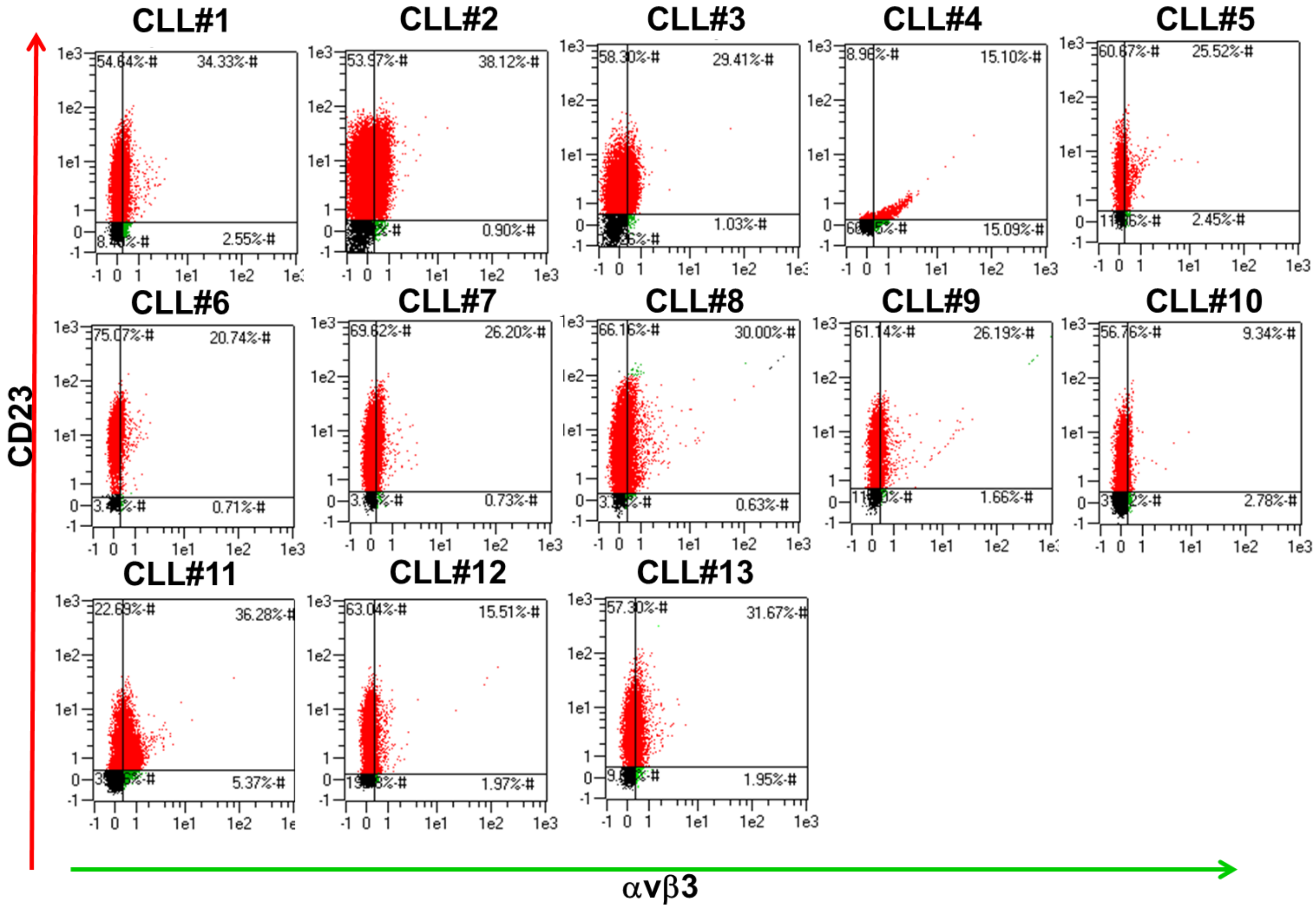

Supplementary Figure S2: αvβ3 is expressed in B lymphocyte cells from CLL patients. Flow-cytometry analyses of αvβ3 (FITC-labeled) and CD23 (PE-labeled) in cells from a selected group of the CLL study cohort (n=13). Percentage of gated cells is presented.

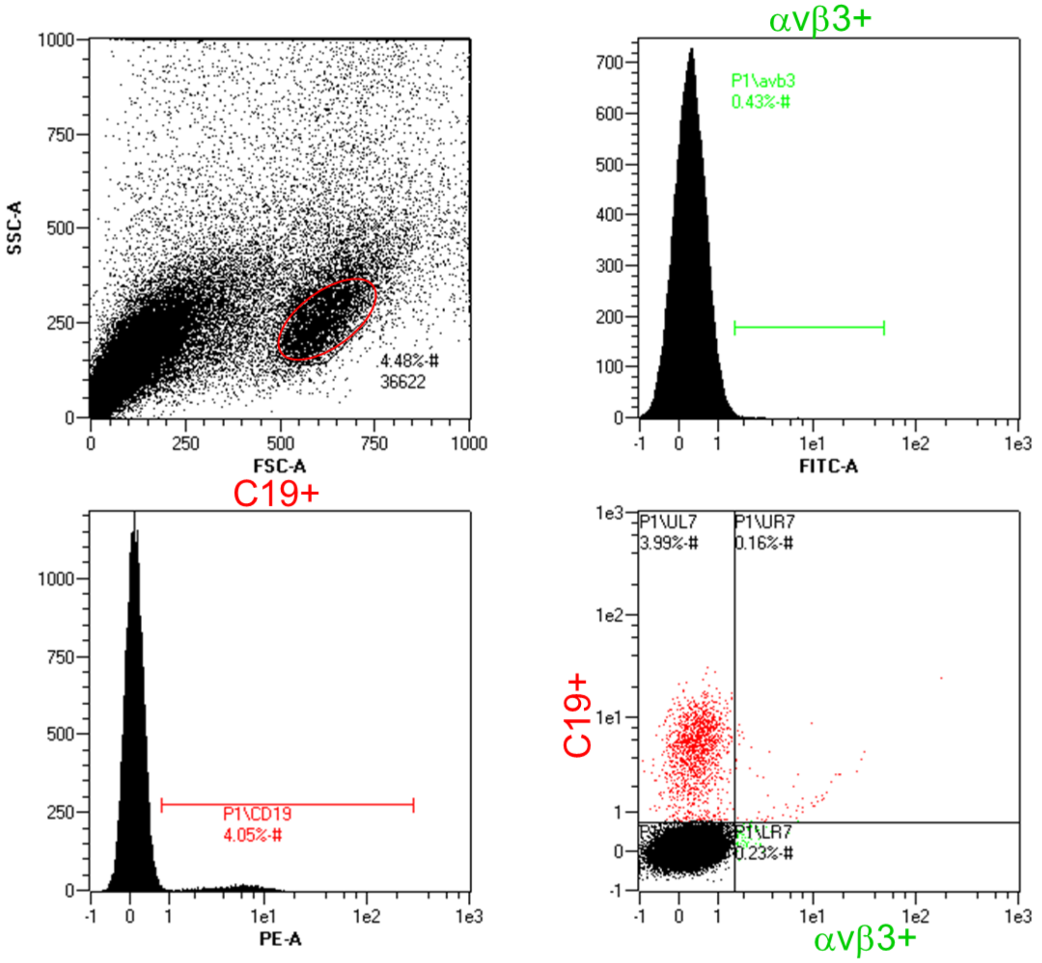

Supplementary Figure S3: αvβ3 is absent in normal B lymphocytes. Flow-cytometry analyses of αvβ3 (FITC-labeled) and CD19 (PE-labeled) in mononuclear cells from a pool of healthy individuals (n=3)

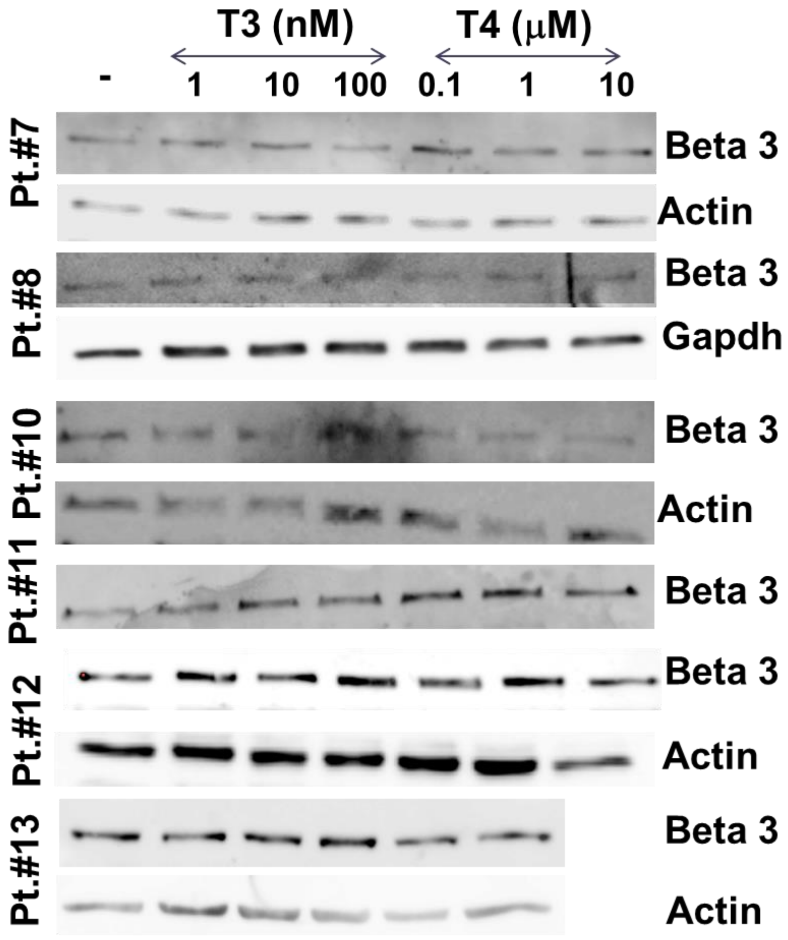

Supplementary Figure S4: T3/T4-induced β3 integrin in CLL cells. Western blots analyses of β3 integrin monomer performed on whole-cell lysates of CLL cells from a selected group of the CLL study cohort (n=6) after 30 minutes T3/T4 treatments over a dose range. Actin beta or GAPDH were used as loading controls. Representative blot of two repeats is presented

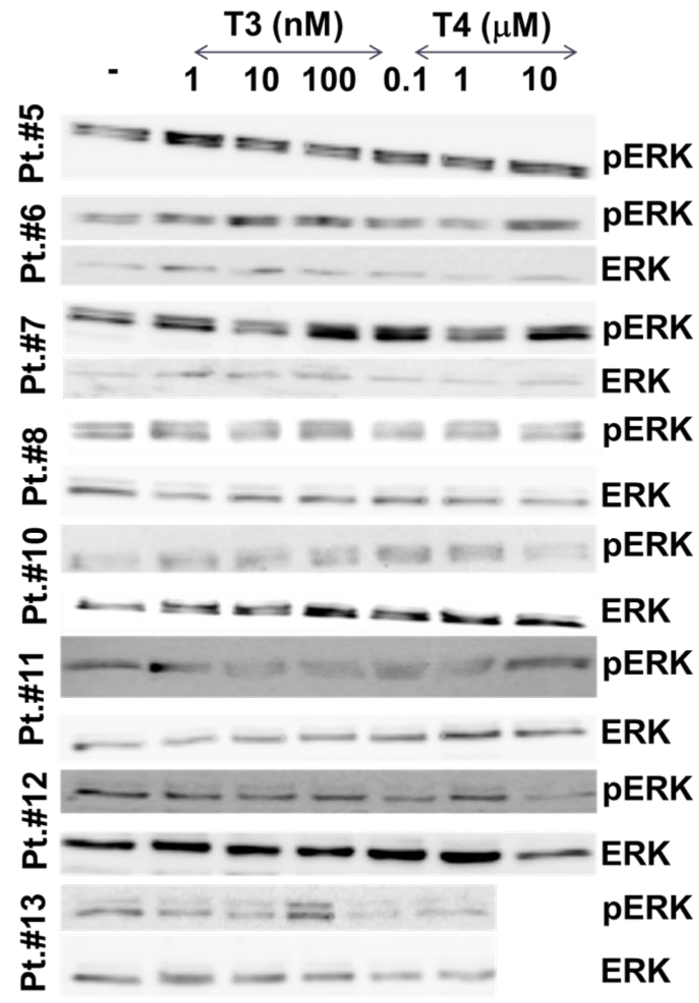

Supplementary Figure S5: T3/T4-induced ERK activation in CLL cells. Western blots analyses of pERK and total ERK performed on whole-cell lysates of CLL cells from a selected group of the CLL study cohort (n=8) after 30 minutes T3/T4 treatments over a dose range. Representative blot of two repeats is presented

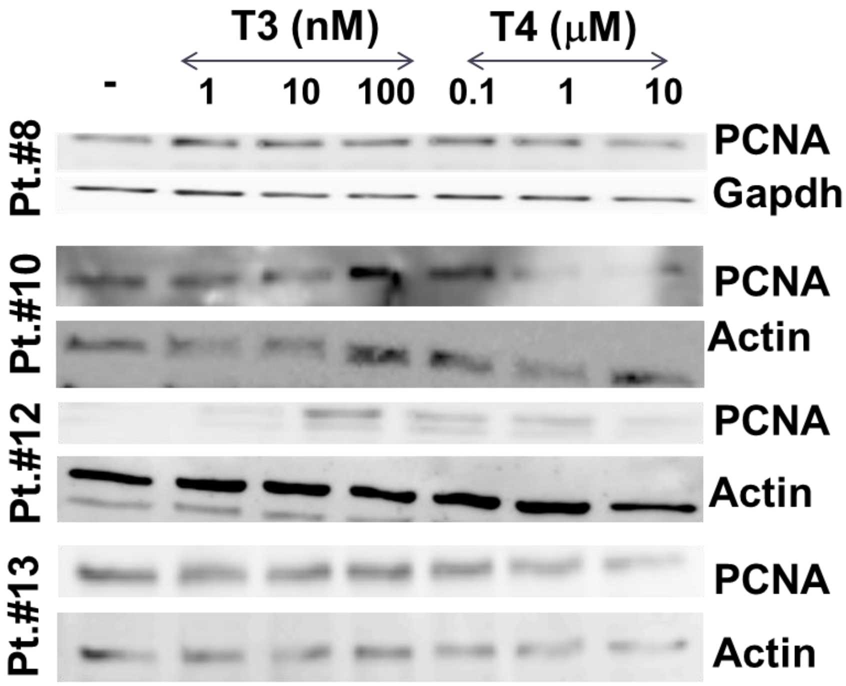

Supplementary Figure S6: T3/T4-induced PCNA activation in CLL cells.

Western blots analyses of PCNA performed on whole-cell lysates of CLL cells from a selected group of the CLL study cohort (n=4) after 30 minutes T3/T4 treatments over a dose range. Actin beta or GAPDH were used as loading controls. Representative blot of two repeats is presented
